# Vitamin E-Enhanced Liners in Primary Total Hip Arthroplasty: A Systematic Review and Meta-Analysis

**DOI:** 10.1155/2021/3236679

**Published:** 2021-12-06

**Authors:** Qian-Yue Cheng, Bin-Fei Zhang, Peng-Fei Wen, Jun Wang, Lin-Jie Hao, Tao Wang, Hui-Guang Cheng, Ya-Kang Wang, Jian-Bin Guo, Yu-Min Zhang

**Affiliations:** ^1^Department of Joint Surgery, Honghui Hospital, Xi'an Jiaotong University, No. 555 Youyi East Road, Beilin District, Xi'an, 710054 Shaanxi Province, China; ^2^Xi'an Medical University, Hanguang North Road, Beilin District, Xi'an, 710068 Shaanxi Province, China

## Abstract

**Objective:**

Adding vitamin E to highly cross-linked polyethylene liners is frequently performed in clinical practice, aiming at reducing liner wear, increasing liner survival, and delaying revision surgery. This study is aimed at evaluating the revision rate, total femoral head penetration, and postoperative clinical function of highly cross-linked polyethylene liners with and without vitamin E in total hip arthroplasty.

**Methods:**

We conducted a systematic literature search to identify the use of highly cross-linked vitamin E liners compared to other liners in patients who received total hip arthroplasty (THA) before April 2021. The study quality assessment and data collection were conducted by two independent reviewers. Studies were artificially grouped, and vitamin E-enhanced liners (VE-PE) were compared with vitamin E-free liners (non-VE-PE). Analyses were executed using Review Manager version 5.4.1.

**Results:**

From the preliminary screening of 568 studies, fourteen studies met the research criteria. Compared to non-VE-PE, using VE-PE reduced the all-cause revision rate (odds ratio = 0.54; 95% confidence interval (CI) 0.40, 0.73; *P* < 0.0001). The total femoral head penetration of the VE-PE was lower than that of the non-VE-PE (mean difference = −0.10; 95% CI -0.17, -0.03; *P* = 0.007). However, there was no difference in clinical function, including the Harris Hip Score and EuroQol Five-Dimension Questionnaire scores.

**Conclusion:**

Compared to the liners without vitamin E, the addition of vitamin E to liners could reduce the all-cause revision rate by approximately 46% in the short-term follow-up. In addition, even though addition of vitamin E could also slow down femoral head penetration, there is no contribution to clinical function.

## 1. Introduction

Total hip arthroplasty (THA) is one of the most successful orthopedic surgeries, and it has brought immeasurable relief to patients with hip joint disease around the world [1]. However, postoperative complications are the biggest obstacle to patient recovery from primary hip replacement. The most important concern is that the duration of the prosthesis directly leads to the timing of revision surgery [2]. One of the main reasons leading to revision surgery is aseptic loosening.

Earlier studies have found that the wear rate of traditional ultrahigh molecular weight polyethylene liners can directly lead to aseptic loosening of the prosthesis, thus promoting earlier revision surgery [3]. In order to combat the disadvantages of ultrahigh molecular weight polyethylene, in the late 1990s, scientists discovered highly cross-linked polyethylene [4], but a new problem arose; the manufacturing of highly cross-linked polyethylene (HXLPE) liners generated free radicals during the sterilization and irradiation process [5]. Free radicals cause damage to the internal polymer structure of the liner and accelerate oxidation. HXLPE liners can be remelted and annealed in the process to remove free radicals. Some scholars have pointed out, however, that this method cannot eliminate the possibility of internal oxidation [6–8].

With more in-depth research, it was found that vitamin E is a better antioxidant that can block the oxidation chain reaction of free radicals and specifically improve the processing disadvantages of HXLPE [9, 10]. In vitro studies have shown that injecting vitamin E into HXLPE not only leads to a lower wear rate but also eliminates free radicals generated by irradiation [11]. Vitamin E injected into high-molecular-weight polyethylene can produce stable oxidation resistance, improve the mechanical properties of the cushion, and prolong the lifespan of the prosthesis [12].

Nevertheless, a substantial amount of clinical data and evidence to verify the effectiveness of vitamin E enhancing in polyethylene liners (VE-PE) is lacking. A meta-analysis by Wyatt et al. [13] in 2019 proposed that vitamin E-enhanced liners have advantages in terms of wear. However, the study only included five randomized controlled trials (RCTs). After that, multiple relevant studies were completed and published. Thus, the main purpose of this updated review was to compare the clinical efficacy of VE-PE and non-VE-PE liners, focusing on the revision rate and head penetration rate.

## 2. Methods

This review was based on previously published studies, and study approval was waived by the Institutional Review Board. Inclusion criteria were as follows: (1) study type: RCTs or observational studies (OSs), including cohort studies and case-control studies; (2) participants: adult patients with unilateral or bilateral osteoarthritis, traumatic arthritis, or avascular necrosis of the femoral head requiring initial THA; (3) intervention measures: patients who underwent THA were divided into two groups, patients with vitamin E added to the VE-PE group and patients without vitamin E added to the non-VE-PE group; and (4) outcomes: all-cause revision rate, total femoral head penetration rate, Harris Hip Score (HHS), and the EuroQol Five-Dimension Questionnaire (EQ-5D). Patients undergoing revision THA surgery were excluded.

### 2.1. Search Strategy

According to the Cochrane Handbook for Systematic Reviews of Interventions, we searched PubMed, Embase, and the Cochrane Library using the following terms: “replacement, arthroplasty, hip”, “hip replacement”, “hip arthroplasty”, “vitamin E”, and “liner”. The search period was from the creation of the database to April 2021. There were no other restrictions for the search process.

### 2.2. Data Extraction and Quality Analysis

Two professional reviewers conducted data extraction and quality evaluation of the full text of the included articles. The Cochrane Handbook for Systematic Reviews of Interventions was used to evaluate the quality of the included RCTs, including sufficient random sequence generation, allocation concealment, blinding, incomplete result data, selective reporting bias, and other biases. The Newcastle-Ottawa Scale was used to evaluate the quality of OSs [14], including population selection, population comparability, exposure assessment, and outcome assessment.

### 2.3. Statistical Analysis

Review Manager (RevMan, The Cochrane Collaboration, London, United Kingdom) version 5.4.1 was used for the statistical analysis. We used odds ratio (OR) and mean difference (MD) to present count data and continuous variables and calculated the 95% confidence interval (CI). When the statistical heterogeneity between studies was low (*P* > 0.1, *I*^2^ < 50%), the fixed-effects model was used for analysis. When the statistical heterogeneity between studies was high (*P* < 0.1, *I*^2^ > 50%), then the possible sources of heterogeneity and possible interference factors were analyzed [15]. If there was only statistical but no clinical heterogeneity, a random effects model was used to pool the data. A *P* value < 0.05 was used to indicate statistical significance.

## 3. Results

### 3.1. Literature Search

**Figure 1:**
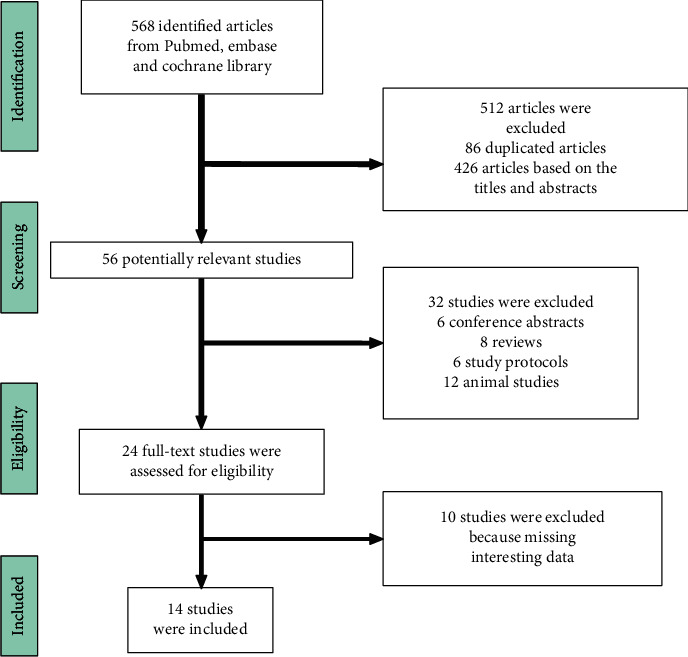
Flowchart of included studies.

The initial database search yielded 568 studies; after the preliminary screening, 512 articles were excluded by reading the titles and abstracts. From the remaining 56 studies, the reviewers excluded abstracts, reviews, protocols, and animal studies based on the inclusion and exclusion criteria. Finally, 14 studies were included, containing 11 RCTs and 3 case-control studies [16–29]. The flowchart is shown in Figure 1.

### 3.2. Baseline Information of the Included Studies

The 14 included studies involved 7560 patients at baseline, of which 2130 were from the 11 RCTs, and 5430 patients were from the three OSs, in multiple medical centers. At study completion, 7523 patients were included in the data analysis (3849 patients in the VE-PE group and 3674 in the non-VE-PE group). The specific baseline information of the included studies is shown in Table 1.

### 3.3. Quality Assessment

Regarding the RCTs, five studies did not clearly indicate the specific process of blinding and random allocation [17, 19–21, 25], two studies [16, 27] used sealed hidden envelopes for allocation but did not mention specific blinding, and four studies used computer randomized allocation. Study bias was low in these RCTs [22–24, 26]. In the quality assessment of OSs using the Newcastle-Ottawa Scale, the scores for the studies conducted by Sillesen et al. [28], Galea et al. [18], and Hemmilä et al. [29] were 6, 8, and 5, respectively.

Among the 14 included studies, most of the articles used Roentgen Stereogrammetric Analysis (RSA) imaging software to measure the wear. There was basically no difference in the data for the prosthesis offset and the change of the cup angle. Most of the postoperative evaluation indexes obtained were HHS or Merle d'Aubigné and Postel scores, while quality of life was assessed using the University of California at Los Angeles hip rating scale, the Numerical Rating Scale, EQ-5D, and Short Form Health Survey. We extracted data pertaining to revision and femoral head penetration rates, HHS, and EQ-5D for comparative analyses.

### 3.4. All-Cause Revision Rate

**Figure 2:**
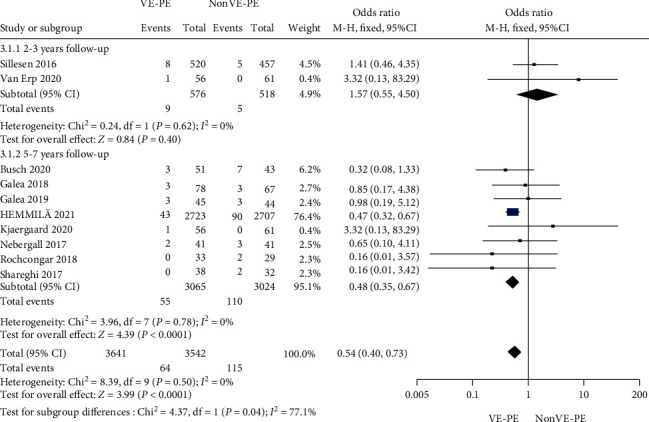
Forest plot of revision rate.

Nine of the 14 studies reported revision rates caused by varied reasons. In particularly, Hemmilä et al. [29] collected data from multiple medical centers for comparison. The revision rates were not reported in the remaining studies because the follow-up time was short or because of surgical success. As shown in Figure 2, VE-PE could decrease the revision rate (OR = 0.54; 95% CI [0.40, 0.73]; *P* < 0.0001; *I*^2^ = 0%). Revision due to aseptic loosening was rare, and most revisions were due to dislocations and infections.

We also set up a subgroup analysis for different follow-up periods. Both the 2-3 years' and 5-7 years' subgroups showed heterogeneity *I*^2^ = 0%. However, we found that there was no difference in revision rate in the 2-3 years' follow-up subgroup (OR = 1.57, 95% CI [0.55, 4.50], *P* = 0.40), while in the 5-7 years' follow-up subgroup, the use of VE-PE was found to decrease the revision rate (OR = 0.48, 95% CI [0.35, 0.67], *P* < 0.0001, Figure 2). In the sensitivity analysis, there was no significant change after selecting individual studies one by one, indicating that the results were stable.

### 3.5. Radiographic Results

#### 3.5.1. Total Femoral Head Penetration

**Figure 3:**
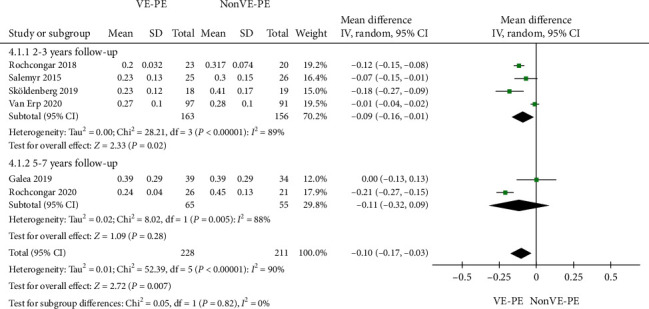
Forest plot of femoral head penetration rate.

Among the included studies, Busch et al. [21] used computer-assisted design software to process images, Van Erp et al. [22] used PACS View Pro-X, and Galea et al. [17] and Sillesen et al. [28] used Martell Hip Analysis Suite software. The study of Hemmilä et al. [29] mainly focused on the revision and survival rate of the prosthesis, and there was no specific report on image processing methods. All other studies used RSA. We found a high level of heterogeneity among studies (*I*^2^ = 90%) and that the total femoral head penetration in the VE-PE group was slower than that in the non-VE-PE group (MD = −0.10, 95% CI [-0.17, -0.03], *P* = 0.007) (Figure 3). VE-PE was therefore superior to non-VE-PE in terms of total femoral head penetration.

However, after analyzing subgroups according to the follow-up period, it was found that VE-PE was superior to non-VE-PE at 2-3 years of follow-up (MD = −0.09, 95% CI [-0.16, -0.01], *P* = 0.02), but there was no difference at 5-7 years of follow-up (MD = −0.11, 95% CI [-0.32, 0.09], *P* = 0.28). The heterogeneity was mainly derived from statistical heterogeneity. After excluding individual studies one by one, we found that the results did not change significantly.

We also compiled the mean femoral head penetration data of resting studies. Since most of the literature adopted the median (interquartile range (IQR)) data presentation, the data reports were not complete. We only conducted a descriptive analysis of this data, which can be found in Table 2. The result showed the penetration rate was lower in the VE-PE group than in the non-VE-PE group.

### 3.6. Function

**Figure 4:**
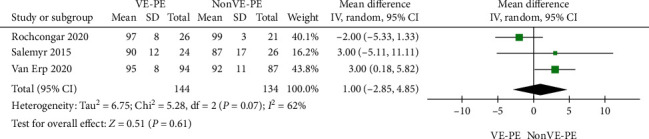
Forest plot of Harris Hip Score.

Eight studies used the HHS to assess postoperative clinical efficacy. Five papers used median and IQR, as shown in Table 3, and three papers were used for the pooled analysis. We found that there was no difference between the VE-PE and non-VE-PE groups (MD = 1.00; 95% CI [-2.85, 4.85], *P* = 0.61) (Figure 4), and the conclusions from the descriptive analysis indicated that there was no difference in postoperative functional recovery between the two groups (Table 3).

We also obtained the EQ-5D scores from five studies, which were expressed in the form of median and IQR.  Table 4 demonstrates that there was no significant difference between groups in any study.

### 3.7. Publication Bias

**Figure 5:**
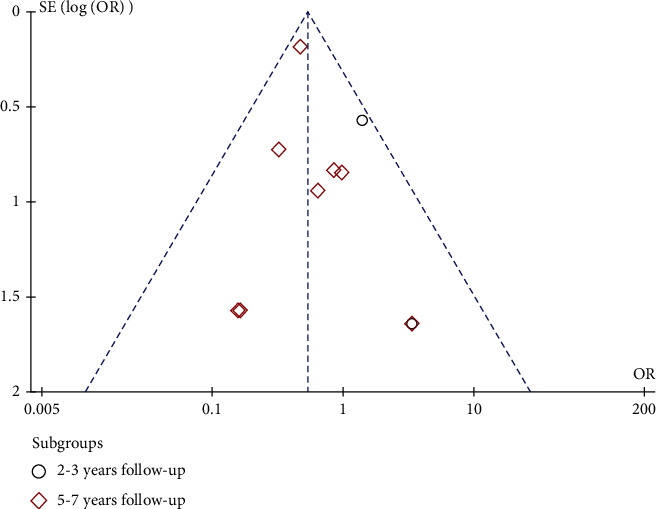
Funnel plot of publication bias.

We made a funnel chart of revision to evaluate publication bias in Figure 5. There was the possibility of publication bias due to inferior symmetry.

## 4. Discussion

Currently, the benefits of THA are recognized by doctors and patients, and technological developments continue to arise, with manufacturers constantly trying to increase the lifespan of prostheses [30, 31]. In this regard, the antioxidant properties of vitamin E are valued as a method to improve the liner coating and reduce wear; this method has been used clinically in a wide range of medical centers [29].

Our meta-analysis has showed that VE-PE provides obvious advantages in revision rate at 2-7 years of follow-up. In addition, VE-PE may slow down the total femoral head penetration compared with the non-VE-PE at 2-3 years of follow-up, but not at 5-7 years. These femoral head penetration outcomes are similar to the findings of in vitro studies [11], further confirming the advantages of VE-PE. Nevertheless, although there was an advantage in wear data, there was no difference in clinical function. The actual experience of patients shows that the benefits of using VE-PE are not as obvious as expected [32]. This was roughly the same conclusion of a previous meta-analysis [13]; the current results may be more robust due to the larger number of studies and patients.

Compared to traditional metal heads, ceramic femoral heads have a very low revision rate. A large-scale national joint registration analysis [33] indicated that lining ceramic heads with VE-PE may further reduce the revision rate. In our analysis, it was found that the revision rate of the VE-PE group was reduced by 46% (1-0.54) compared with that of the non-VE-PE group. The number of revision surgeries due to liner wear was also extremely reduced. We included all factors, including aseptic loosening, dislocation, trauma, and infection in the repair rate, among which the number of dislocation and infection were the most frequent factors leading to revision surgery. Although this encouraging result is derived from the comparison in Figure 1, it does not directly represent an absolute advantage of adding vitamin E. It is worth mentioning that, regardless of the use of vitamin E, all studies measured the migration and osteolysis of the acetabular cup, except for cases that required revision.

In addition, comparing the annual average femoral head penetration rate, it was found that VE-PE could slow down the total femoral head penetration compared with non-VE-PE at 2-3 years of follow up, but not at 5-7 years. This inconsistency may be related to the length of the follow-up time. Over time, the concentration of vitamin E decreases, thus diminishing its effect. This shows that the advantages of VE-PE are not as significant as expected.

As for the clinical function, we used HHS and EQ-5D scores to assess postoperative clinical efficacy and found that vitamin E does not influence the postoperative functional recovery. This result is in line with the role of vitamin E, which blocks the oxidation chain reaction of free radicals in bear [9, 10] but does not improve hip function.

We also assessed publication bias through a funnel plot. Due to the inferior symmetry, it was indicated that there was the possibility of publication bias. We consider that studies focusing on the short-term revision might be lost.

This study had several limitations. Firstly, we included three OSs since these studies, which had relatively high quality and a sufficiently large sample size, were relevant to the current review. Secondly, the problem we encountered in the data extraction process was that the presentation of the data in the original text was not sufficiently uniform to allow us to compare multiple articles horizontally. Thirdly, prosthesis wear is affected not only by the liner but also by the size and material of the femoral head. In addition, the follow-up time was relatively large in the articles. The study of Hemmilä et al. [29] has a follow-up time of up to 11 years. The follow-up time in the studies Van Erp et al. [22], Salemyr et al. [25], and Sköldenberg et al. [16] is shorter, at only 2 years. The average follow-up period of the 14 studies was 4.3 years (4 years in RCTs and 5.3 years in OSs). These limitations indicate that further higher-quality RCTs, with longer follow-up, unified measurement standards, and unified prosthesis types, are needed.

## 5. Conclusion

Compared to the liners without vitamin E, the addition of vitamin E could reduce the revision rate of all causes by approximately 46% in the short-term follow-up. In addition, even though the addition of vitamin E could also slow down femoral head penetration, there is no contribution to clinical function.

## Figures and Tables

**Table 1:** Baseline information of included studies.

Study	Years	Type of study	Grouping	Sample size	Age	Inclusion criteria	Surgery approach	Observation index	Follow-up
Rochcongar et al. [24]	2020	RCT	HXLPE/VitE	33	61 ± 6.5	Primary or secondary osteoarthritis or osteonecrosis	Not reported	Liner wear, femoral head penetration, osteolysis, prosthesis loosening, cup angle, HHS, MAP	5 y
			UHMWPE	29	61 ± 7.8				
Kjærgaard et al. [20]	2020	RCT	vE-PE	53	64 (56-67)	Primary osteoarthritis, 40-70 years of age	Posterolateral approach	Liner wear, femoral head penetration, osteolysis, prosthesis loosening, cup angle, HHS, UCLA, EQ-5D, SF-36	5 y
			XLPE	63	62.5 (54-66)				
Sköldenberg et al. [16]	2019	RCT	VE	21	67 ± 5	Primary osteoarthritis	Posterolateral approach	Liner wear, femoral head penetration, osteolysis, prosthesis loosening, cup angle, bone cement quality, HHS, UCLA, EQ-5D, SF-36	2 y
			ArComXL	21	67 ± 4				
Busch et al. [21]	2020	RCT	UHMWPE-XE	51	Not reported	Primary osteoarthritis	According to the habit of the surgeon	CAD, liner wear, femoral head penetration, osteolysis, prosthesis loosening, cup angle	5 y
			UHMWPE-X	43					
Nebergall et al. [19]	2017	RCT	HXLPE/VitE	32	67 (43-76)	Primary osteoarthritis, 25-75 years of age	Posterolateral approach	Liner wear, femoral head penetration, osteolysis, prosthesis loosening, cup angle, HHS, UCLA, EQ-5D, SF-36, VAS	5 y
			ArComXL	35	65 (40-73)				
Scemama et al. [26]	2017	RCT	VE-PE	38	67 (32-74)	Primary or secondary arthritis, age < 75	Posterolateral approach	Liner wear, femoral head penetration, osteolysis, prosthesis loosening, cup angle, Merle d'Aubigné hip score	3 y
			UHMWPE	36	66 (49-75)				
Galea et al. [17]	2019	RCT	VEPE	44	66.1 ± 6.5	Primary osteoarthritis, 25-75 years of age	Posterolateral approach	Liner wear, femoral head penetration, osteolysis, prosthesis loosening, cup angle, HHS, UCLA, EQ-5D, SF-36, NRS	7 y
			ModXLPE	45	62.6 ± 8.3				
Rochcongar et al. [23]	2018	RCT	HXLPE/VitE	33	61 ± 6.5	Primary or secondary osteoarthritis or osteonecrosis, 18-75 years of age	Not reported	Liner wear, femoral head penetration, osteolysis, prosthesis loosening, cup angle, HHS, MAP	3 y
			UHMWPE	29	61 ± 7.8				
Shareghi et al. [27]	2017	RCT	HXLPE/VitE	38	Not reported	Primary osteoarthritis, 25-75 years of age	Not reported	Femoral head penetration, cup migration, femoral stem migration, HHS	5 y
			ArComXL	29					
Van Erp et al. [22]	2020	RCT	HXLPE/VitE	102	66 ± 5	Ages 20-85, primary osteoarthritis, osteoarthritis, rheumatoid arthritis, avascular necrosis of the femoral head or trauma	According to the habit of the surgeon	Liner wear, femoral head penetration, osteolysis, prosthesis loosening, cup angle, HHS, NRS	2 y
			UHMWPE	97	65 ± 5				
Salemyr et al. [25]	2015	RCT	VE-HXLPE	25	62 ± 6	Primary osteoarthritis, 40-70 years of age	Posterolateral approach	Liner wear, head penetration, HHS, EQ-5D	2 y
			HXLPE	26	62 ± 5				
Galea et al. [18]	2018	OS	VEPE	136	59.8 ± 10.3	Primary osteoarthritis	According to the habit of the surgeon	Liner wear, femoral head penetration, osteolysis, prosthesis loosening, cup angle, HHS, VAS, EQ-5D, SF-36	5 y
			ModXLPE	57	60.8 ± 8.2				
Sillesen et al. [28]	2016	OS	E-XLPE	520	Not reported	Primary or secondary osteoarthritis, 25-75 years of age	Posterolateral approach, direct lateral approach, or direct anterior approach	Liner wear, femoral head penetration, osteolysis, prosthesis loosening, cup angle, HHS, UCLA, EQ-5D, SF-36, VAS	3 y
			ArComXL	457					
Hemmilä et al. [29]	2021	OS	VE-HXLPE	2723	67 ± 10	Primary hip replacement	According to the habit of the surgeon	Revision, revision for loosening of the cup, osteolysis, liner wear, or liner breakage	5 y
			HXLPE	2707	64 ± 9				11 y

**Table 2:** Mean femoral head penetration rate (mm/year).

Study	VE-PE	Non-VE-PE	Conclusion
Sillesen et al. [28]	0.005	0.027	No difference
Shareghi et al. [27]	0.04	0.08	No difference
Kjærgaard et al. [20]	-0.006	0.005	VE-PE better
Busch et al. [21]	0.024	0.023	No difference
Galea et al. [18]	0.02 (mental)	0.02 (ceramic)	VE-PE better
	0.00 (ceramic)	0.02 (ceramic)	
Van Erp et al. [22]	0.046	0.056	VE-PE better
Rochcongar et al. [23]	0.020	0.058	VE-PE better
Rochcongar et al. [24]	0.020	0.060	VE-PE better
Scemama et al. [26]	0.008	0.133	VE-PE better

**Table 3:** Median (IQR) Harris Hip Score.

Study	VE-PE	Non-VE-PE	Conclusion
Sillesen et al. [28]	91.19 (23 to 100)	90.08 (27 to 100)	No difference
Galea et al. [18]	93 (88 to 98)	96 (91 to 100)	No difference
Galea et al. [17]	94 (88 to 100)	97 (91 to 100)	No difference
Nebergall et al. [19]	93 (88 to 98)	97 (93 to 100)	No difference
Shareghi et al. [27]	95 (35 to 100)	94 (36 to 100)	No difference

**Table 4:** Median (IQR) EQ-5D.

Study	VE-PE	Non-VE-PE	Conclusion
Galea et al. [18]	0.8 (0.7 to 1.0)	1.0 (0.7 to 1.0)	No difference
Galea et al. [17]	1.0 (0.7 to 1.0)	1.0 (0.8 to 1.0)	No difference
Nebergall et al. [19]	1.0 (0.7 to 1.0)	1.0 (0.8 to 1.0)	No difference
Salemyr et al. [25]	0.92 ± 0.14	0.87 ± 0.22	No difference
Sillesen et al. [28]	0.86 (0 to 1.0)	0.83 (0 to 1.0)	No difference

## Data Availability

Data sharing is not applicable to this article as no datasets were generated or analyzed during the current study.

## Abbreviations

THA: Total hip arthroplasty
HXLPE: Highly cross-linked polyethylene
VE-PE: Vitamin E in polyethylene
RCT: Randomized controlled trial
OS: Observational study
HHS: Harris Hip Score
EQ-5D: EuroQol Five-Dimension Questionnaire
OR: Odds ratio
MD: Mean difference
95% CI: 95% confidence interval
RSA: Roentgen Stereogrammetric Analysis.
